# Operations on the Liver

**Published:** 1898-03-26

**Authors:** Leonard A. Bidwell

**Affiliations:** Senior Assistant-Surgeon to the Hospital


### Medical Progress and Hospital Clinics.

[*The Editor will be glad to receive offers of co-operation and contributions from members of the profession. All letters should be addressed to* The Editor, at the Office, 28 & 29, Southampton Street, Strand, London, W.C.]

### OPERATIONS ON THE LIVER.

A Postgraduate Lecture, delivered at the West London Hospital by Leonard A. Bidwell, F.R.C.S., Senior Assistant-Surgeon to the Hospital.

(*Continued from page* 429.)

**Injury.**—The chief symptoms in cases of injury to the liver requiring operation are: (1) Collapse; (2) pain situated in the right hypochondrium; (3) vomiting, often bile-stained; (4) occasionally jaundice; and (5) the presence of bile in the urine.

The nature of the force producing the injury and its mode of application are also of importance in arriving at the diagnosis.

When an operation is decided on the liver is exposed by an incision in the right linea semilunaris, and a search made for the rupture. If the bleeding is still going on, after removing the blood clots the wound in the liver should be closed with thick silk sutures, which are passed deeply on each side of the rupture and tied tightly. In the case of small lacerations near to the surface, or of those so deeply placed that no suture can be inserted, a pack of iodoform gauze should be passed into the wound in the liver and brought out of the abdominal incision. It is astonishing how little pressure is needed to stop even free bleeding from the liver. Any portion which is completely detached, as often happens in cases of rupture of the liver from a kick, should of course be removed. Suture of injuries of the liver has yielded satisfactory results in experiments on animals. In many cases the rupture is too extensive to be dealt with surgically, since either death is almost instantaneous, or the shock of the injury is too great to permit of an operation.

In one of the following cases death was almost instantaneous, and in the other the kidney also was ruptured.

Case IV.: A man aged 43 was kicked by a horse in the right hypochondrium, fracturing the eighth, ninth, and tenth ribs. He complained of great pain, but walked a short distance and then fainted. He was brought into the hospital dead two hours after the accident. At the post-mortem examination the abdomen was full of blood-clot, and nearly the whole of the right lobe of the liver was completely detached. No other organ was injured.

Case V.: A man, aged 32, was admitted into this hospital on May 22nd, 1896, having been run over by a four-wheel wagon loaded with hay. He was in a state of collapse, and had severe pain in the right hypochondriac and lumbar regions. Shortly after admission he became blanched, restless, and vomited blood; dulness and swelling appeared in the right flank. About seven hours after the accident I opened the abdomen in the middle line, and found a rent on the under surface of the liver, and the gall-bladder was detached. There was no active bleeding from the rent in the liver, but as blood welled up from the right loin the right kidney was examined and found to be completely torn in half. It was therefore removed, and the abdominal cavity was irrigated with saline solution to remove the blood-clots. Saline solution was also infused into the basilic vein. The patient never rallied, but died five hours later. There had been no further bleeding into the peritoneal cavity.

**Displacement of the Liver.**—In this condition the whole liver descends below the ribs and forms a large tumour, which can be pushed back under the ribs; the chief symptoms may be due to the dragging on the pylorus, which causes dilatation of the stomach. In order to make certain whether any tumour in the right hypochondrium is connected with the liver, Fremissen's test should be employed; this consists in injecting air per anum to distend the transverse colon, rendering it tympanitic to percussion; if the swelling be above the distended colon it is associated with the liver, but if below it is connected with some other organ, such as a cystic kidney. A moveable liver can be fixed by the operation of hepatopexy. An incision is made from the eleventh rib along the costal arch, and the peritoneum opened, the liver is then replaced, and its anterior edge is sutured to the upper lip of the abdominal incision with strong silk sutures. The wound is then closed. This operation has been followed by good results, and there does not appear to be any risk of the recurrence of the trouble.

**Tumours.**—New growths of the liver which are capable of removal are not numerous, and an operation is only justifiable when the growth is primary, is of small extent, and no secondary nodules have formed. Angiomata, adenomata, and carcinomata have all been successfully removed. The operation is called hepatectomy, and can be performed in several ways. After exposing the surface of the liver by an incision over the tumour, the tumour may be removed in the following ways: (*a*) The portion of the liver containing the tumour is fixed in the wound, and a strong ligature is applied to strangulate the growth, which sloughs off after a few days. (*b*) The liver is simply fixed in the wound and a gauze pack is inserted; after adhesions have formed the tumour is removed with a knife and the wound plugged. (*c*) The tumour is removed with the knife without fixing the liver in the wound, and the gap in the liver is closed with stout silk sutures. (*d*) A wedge of liver is removed with the tumour, and the edges are brought together with silk sutures.

There is no question that the last-named is the best surgical procedure, and it has proved successful in several cases. The bleeding is all arrested by closing the incision in the liver, but if any oozing continue, a plug of gauze can be left in over the wound in the liver.

**Gall-Stones.**—According to Shröder's statistics gall-stones are present in 4·4 per cent. of all males, and in 20 per cent. of all females; their greater frequency in females is supposed to be due to the effects of tight lacing, costal respiration, and pregnancy, since all of those conditions interfere with the movements of the abdomen, and so hinder the proper emptying of the gall-bladder.

Although the character of the pain in hepatic colic is well known to most of us, it may be well to describe it again minutely. At first the patient has slight pain in the upper part of the right hypochondium, together with a sense of fulness and heaviness in that region. Later on, he suffers from paroxysms of acute pain, which start in the region of the gall-bladder, or in a line running from the top of the ninth rib to the umbilicus, the pain often radiates from the umbilicus, and is specially severe at the inferior angle of the right scapula. These pains last several minutes or hours, and are accompanied sometimes by collapse and sometimes by vomiting, which terminate the attack. They may be followed by jaundice, but this is only present when a stone has entered, and has remained some time in the common bile duct. The absence of gall-stones from the fæces is not of any importance, since in many cases no gall-stones pass into the intestine after an attack of colic.

*(To be continued.)*

